# Perspectives of Nursing Teachers on the Use of Microteaching as a Teaching Technique

**DOI:** 10.7759/cureus.83150

**Published:** 2025-04-28

**Authors:** Natasha M Mahajan, Vaishali S Jadhav, Arunima Sreeletha

**Affiliations:** 1 Critical Care, College of Nursing, Bharati Vidyapeeth (Deemed to be University), Navi Mumbai, IND; 2 Medical-Surgical Nursing, College of Nursing, Bharati Vidyapeeth (Deemed to be University), Navi Mumbai, IND; 3 Child Health Nursing, College of Nursing, Bharati Vidyapeeth (Deemed to be University), Navi Mumbai, IND

**Keywords:** clinical teaching, microteaching, nursing teachers, perspective, skills, teaching

## Abstract

Background

Microteaching is a structured instructional approach that enables educators to develop specific teaching skills in a controlled and supportive environment. By delivering concise lesson segments, teachers receive immediate, targeted feedback that fosters self-reflection and rapid improvement. This approach has proven effective in enhancing communication and classroom management, essential skills for both theoretical instruction and clinical practice. This study delves into the perspectives of nursing teachers on microteaching, exploring its practical benefits and challenges in preparing students for the complexities of professional nursing.

Materials and methods

Employing a phenomenological design, the research was conducted in selected nursing colleges in Mumbai and Navi Mumbai among 12 post-graduate educators with a minimum of three years’ teaching experience. Participants were chosen through simple random sampling. In-depth, face-to-face interviews were conducted, and audio recordings ensured accurate data capture.

Results

Demographic data revealed a diverse group, predominantly female, with most holding an MSc degree and extensive teaching experience. The study identified key themes, including difficulties such as time constraints and evaluation challenges, as well as benefits related to enhancing teaching skills, reflective practice, and clarity in topic delivery. Areas for the application of microteaching included classrooms, clinical settings, laboratories, and community environments. It also improved individual teaching techniques, group dynamics, and communication skills.

Conclusion

The study examined the perspectives of nursing teachers on the use of microteaching as a teaching technique, capturing their experiences through qualitative analysis that addressed notable challenges, application areas, benefits, and skill development.

## Introduction

Microteaching is a focused and systematic teaching technique that provides an opportunity for educators to practice and refine their teaching skills in a controlled, supportive environment. It involves the presentation of a small segment of a lesson, typically ranging from 5 to 10 minutes, to a small group of students or peers. This technique allows nursing teachers to improve specific aspects of their teaching, such as communication, instructional strategies, and classroom management, with immediate feedback from observers. The perspective of nursing teachers on the use of microteaching as a teaching method is increasingly relevant in enhancing teaching effectiveness and providing a platform for self-reflection and professional growth. By embracing microteaching, nursing educators can better prepare students for the challenges of clinical practice and ensure high-quality education in the nursing field.

Dixon KA et al. (2015) conducted a qualitative study on the experiences of sessional teachers in a Bachelor of Nursing program at an Australian university. They found that teachers enjoyed teaching and valued their clinical currency, but identified areas needing improvement such as system issues, microteaching and assessment skills, and classroom management [1]. Ralph EG (2014) studied the effectiveness of microteaching among teacher candidates in Canada, concluding that microteaching enhanced teaching competence and confidence, particularly under favorable conditions [2]. Similarly, Shanti R and Lakshmi R (2014) surveyed teachers and postgraduate nursing students about microteaching, finding that most teachers viewed it as an effective method, helping them identify weaknesses and requiring adequate resources. The study revealed that postgraduate teachers conducted microteaching twice a year and often implemented it in clinical settings [3].

A qualitative study by Necmeddin BH and Ismail (2021) examined preservice teachers’ opinions on microteaching courses, with participants reporting that the courses improved their professional skills, classroom management, and experience. Many expressed a desire to take the course again in the future [4]. Additionally, Ayhan C et al. (2019) investigated primary student teachers’ views of microteaching, finding that feedback was valuable for self-evaluation. However, some students felt confident in their questioning skills and considered measurement and evaluation less important, though they acknowledged the importance of developing questioning skills for higher-order thinking [5]. Through our own experiences, the researchers felt the need to explore the views and challenges related to the implementation of microteaching. There is also a need to explore different areas, such as theoretical and clinical settings, where microteaching can be used as an effective teaching technique. The implementation of microteaching enables teachers to adopt discussion-oriented teaching and enhance student learning.

## Materials and methods

This study employed a qualitative research approach using a phenomenological design to explore the perspectives of nursing teachers on the use of microteaching as a teaching technique. It examined the core experiences of nursing teachers regarding the implementation of microteaching in their teaching practices. The study aimed to understand the experiences and views of nurse educators who had implemented microteaching as their teaching style by focusing on their personal narratives. To ensure an objective assessment of the participants' experiences and to provide a thorough understanding of the phenomenon, the researcher used bracketing to set aside personal biases.

The researchers listed council-recognized nursing institutes in Mumbai and Navi Mumbai. Nine colleges met the predefined criteria. Out of these, five colleges were randomly selected using the lottery chit method to ensure an unbiased selection process. Prior permission was obtained from the respective college authorities. The study included 12 (100%) post-graduate nursing teachers who met the inclusion criteria and contributed until data saturation was achieved. Data saturation was determined when investigators received repeated responses from participants. The participants were also selected using a simple random sampling method, specifically the lottery chit method. Individual consent was obtained from each participant. The study was conducted over a period of four months.

**Figure 1:**
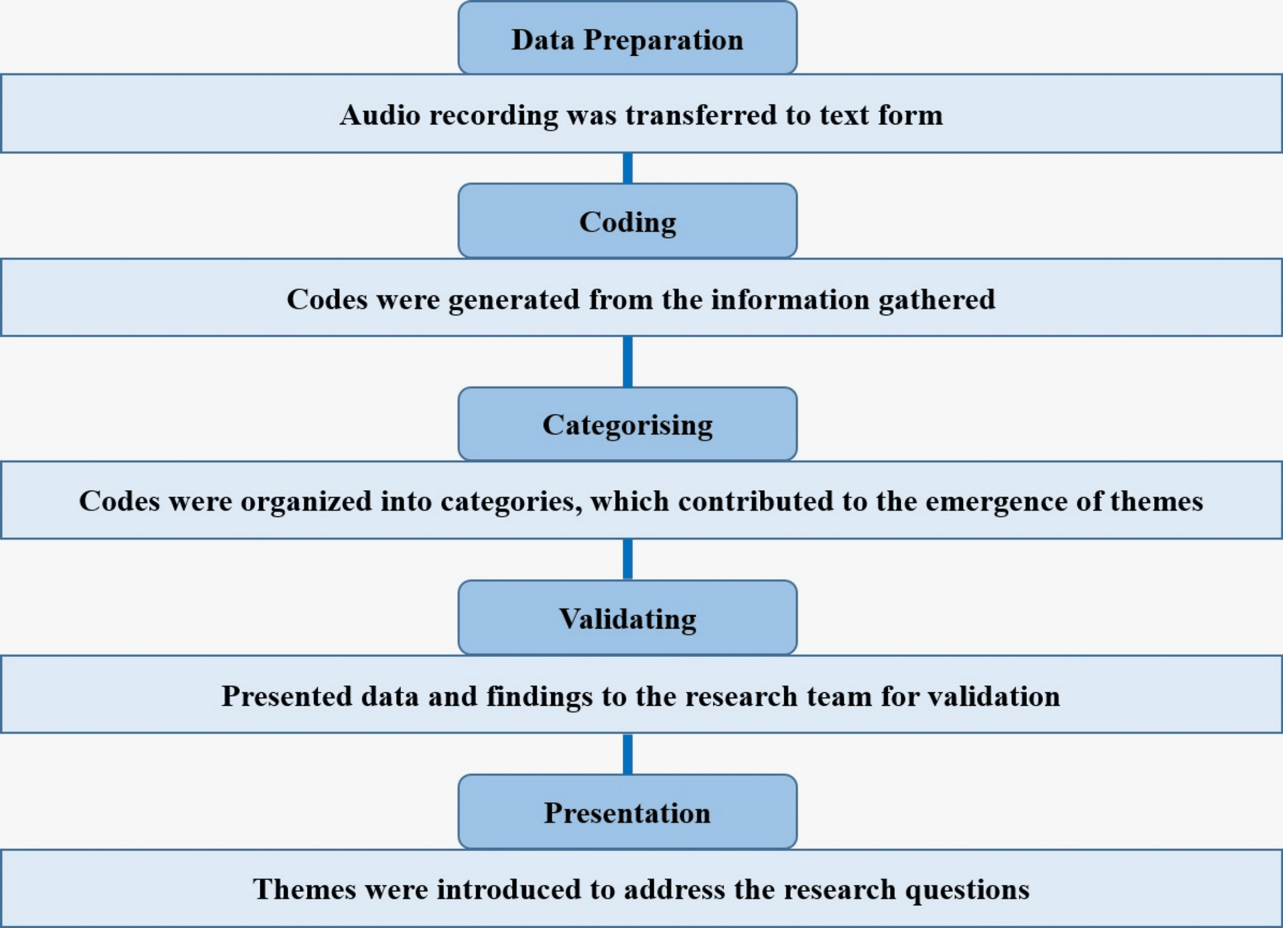
Schematic representation of the process of thematic analysis.

Data was collected through in-depth, face-to-face interviews, which provided participants with the opportunity to share valuable insights into their experiences with microteaching. By audio-recording the interviews, the researchers ensured precise data capture. Data preparation involved transcribing the collected audio recordings into text. Key pieces of information were identified and converted into meaningful codes. These generated codes were grouped into categories, which helped in developing overarching themes. The categorized data and findings were shared with experts in the nursing field to ensure accuracy and reliability. The final themes, subthemes, and codes were presented to effectively address the research questions (Figure 1).

The data gathering process was guided by semi-structured interviews (Appendix 1). The literature served as a guide for the interview questions, which were designed to elicit detailed answers relevant to the study's objective. A system was in place to help participants relax and develop rapport in order to provide a comfortable setting for the interviews. The researcher obtained informed consent and explained the study's goal prior to the interviews. Confidentiality, cultural and ethical considerations, and other delicate aspects of participant interaction were handled professionally by the researcher conducting the interviews. The interviews consisted of four planned open-ended questions. No additional branching questions emerged during the data collection process. The duration of the interviews ranged from 15 to 20 minutes on average. These interviews were audio-recorded with the interviewees' permission, and written notes were also taken.

Direct sociodemographic information was gathered from the subjects. This included age, gender, qualifications, specialization, designation, years of post-graduation teaching experience, years of teaching postgraduate students, and subjects taught. A total of twelve interviews were conducted. Interview recordings from P1 to P12 were labeled using a systematic coding strategy to protect participant privacy. During the interviews, objectivity was upheld to the extent practical, while respecting the viewpoints and ideas expressed through tone, body language, and expressions of interest. Ethical considerations, including permission from nursing colleges and informed consent from participants, were ensured throughout the study.

## Results

The data were analyzed using descriptive and thematic analysis for qualitative data. The qualitative perspective of nursing teachers was analyzed using the Colaizzi phenomenological method. Codes were generated verbatim from 12 (100%) participants based on data saturation. Subthemes were derived from the listed codes, and themes were subsequently generated.

Demographic profile analysis

The demographic data presents the distribution of participants across various categories. Regarding age, 33.33% are below 40 years, 50% are between 40 and 50 years, and 16.67% are above 50. In terms of gender, 16.67% of the participants are male, while 83.33% are female, indicating a higher female representation. Concerning qualifications, a significant majority of participants hold an MSc degree (75%), while 25% have a PhD. In terms of designation, 41.67% of participants are Assistant Professors, 16.67% are Associate Professors, and the remaining 41.67% hold the title of Professor, showing an equal distribution between Assistant Professors and Professors. For work experience, a small proportion (16.67%) have less than 10 years of experience, while 83.33% have over 10 years of experience, reflecting a more experienced participant pool (Table 1).

**Table 1:** Distribution of the sample as per demographic profile.

Demographic Variables	Category	N	%
Age	Below 40	4	33.33
	40-50	6	50
	Above 50	2	16.67
Gender	Male	2	16.67
	Female	10	83.33
Qualification	MSc	9	75
	PhD	3	25
Designation	Assistant Professor	5	41.67
	Associate Professor	2	16.67
	Professor	5	41.67
Experience (Years)	Below 10	2	16.67
	Above 10	10	83.33

Themes depicting the perceptions of teachers on microteaching

**Figure 2:**
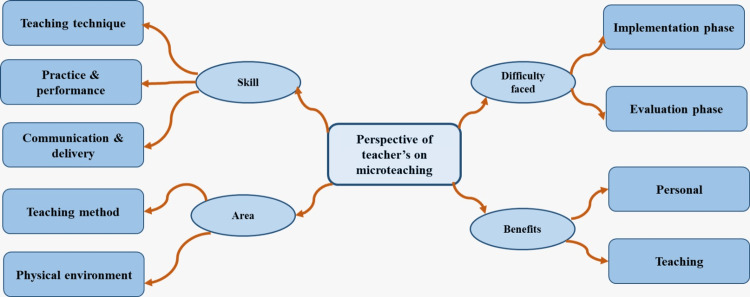
Themes reflecting the perception of teachers regarding microteaching.

The perception of microteaching was categorized into four themes, offering a comprehensive understanding of microteaching as a technique (Figure 2).

Qualitative analysis

The analysis based on the themes is as follows (Table 2):

**Table 2:** Distribution of the perspectives of nursing teachers under themes and subthemes.

Themes	Subthemes	Codes	N	%	Evidence	
Difficulty you faced	Implementation phase	Time constraints	12	100	"It's a short-term method, so we can't cover most of the topics within the given time. When we try to cover the topics, we face difficulty understanding them, that's the main problem we face." (P1)	
		Specific skills	3	25	“Yeah, actually why it happens is, when we assign students some specific technique skills, the students are usually unaware of the particular skills they are going to demonstrate. They get focused on it, and since in microteaching the duration is short, in that short duration they are assigned a specific skill. So we have to be perfect with the skill, first they have to demonstrate the skill they have been assigned, and then they should go for other skills. So that is the thing students get confused about.” (P9)	
		Controlled situations	3	25	"Teaching in this method is quite controlled, and it doesn’t allow for much elaboration on specific topics. The teacher isn’t able to adapt explanations to suit the needs of different students, whether they’re struggling or excelling. The focus remains strictly on the teaching plan rather than diving deeper into the subject matter. Unfortunately, this approach also means that students’ doubts can’t be addressed immediately. It’s really only suitable for use in small classes, as the limitations become more apparent in larger settings." (P12)	
	Evaluation phase	No concrete feedback	2	16.67	"When you ask about difficulties, the first one is that it is time-consuming. It consumes a lot of time because first the student has to prepare a lesson plan, then present, record, and then we have to give feedback. Then, again, we have to take their teaching. So until they get the skills, they have to repeat the cycle. That is the only difficulty, because when we have 10 or 15 students, it becomes difficult for each student to take microteaching as per the procedure or steps. So that is the main difficulty we are facing." (P8)	
		Lack of clarity on evaluation	2	16.67	“One of the difficulties we face in microteaching is evaluating students within a limited timeframe, such as 10, 15, or 20 minutes, during which they demonstrate specific teaching techniques. While they are provided with certain criteria for evaluation, students sometimes do not fully understand these criteria. As a result, they may simply follow a method, like the lecture method, without aligning their approach to the evaluation standards. This lack of clarity can pose a challenge in the process.” (P7)	
Benefits	Personal	Novice teachers	2	16.67	“It is helpful for novice teachers to develop their skills. It will help them make their students understand the topic properly. Since microteaching is of short duration, the skills are very well developed. You can use different types of techniques in it. Basically, it involves investigation, and it is like reply, observe, and again reflect back to explain things.” (P2)	
		Teaching skills	7	58.33	“PG students, they are already adult nurses, so for PG students, it is very useful. We just have to refine their teaching skills. In microteaching, we do not elaborate teaching like in conventional teaching for UGs. From the PG point of view, microteaching is effective. We just have to refresh their knowledge. It gives feedback.” (P3)	
		Confident booster	2	16.67	“This approach is really helpful for improving planning and teaching skills. Since it doesn’t allow for any mixing of unrelated content, the teacher can stay focused on one particular topic, making the session more streamlined. Another great aspect is the feedback it provides, which plays a key role in further improvement. It’s also a confidence booster for the teacher, as they can refine their delivery over time. Plus, because the sessions are short, they can easily be recorded for review or future reference.” (P12)	
		Identifying mistakes	1	8.33	“I think microteaching helps teachers develop and refine their teaching skills. It allows them to recognize their strengths and areas needing improvement by identifying mistakes made during practice. This process provides a valuable opportunity for teachers to enhance their skills through targeted feedback and reflection.” (P4)	
		Reflection	3	25	“From the PG students' teaching point of view, we cover most of the microteaching, so they can recollect, reflect, and understand faster.” (P1)	
	Teaching	Time	4	33.33	“For postgraduate students, the topics may be easier to understand, but for BSc students, it takes a bit more time. Therefore, the time duration for teaching should be slightly extended to accommodate their learning needs.” (P6)	
		Feedback	3	25	“PG students, they are already adult nurses, so for PG students, it is very useful. We just have to refine their teaching skills. In microteaching, we do not elaborate teaching like in conventional teaching for UGs. From the PG point of view, microteaching is effective. We just have to refresh their knowledge. It gives feedback and reflection.” (P3)	
		In-depth focus	2	16.67	“While this can be a limitation, it also has its advantages, as it allows for in-depth focus and clarification of specific topics within any subject. Despite this difficulty, microteaching remains an effective technique for enhancing understanding.” (P5)	
		Clarification of specific topics	2	16.67	“This is basically very helpful in developing the individual skill, and when they go for a seminar or present some bigger topic, then they can make use of all techniques which they practiced during microteaching. So, by this means, it will help them to practice all the techniques, for example, explanation, lecturing, introduction, probing questions. So all techniques or skills which are there, they can accumulate, articulate, and display altogether before the seminar. But during microteaching, individually, they can practice it and improve.” (P9)	
		Enhancing understanding	6	50	“The benefit is that within a short period of time, we can brief students. As well, we can use many AV aids and innovative techniques in microteaching, so it helps students to better understand. It also helps because we use many AV aids, it helps them remember things for a longer period of time. Microteaching also helps them, because presently students don’t want to listen to long lectures. It’s one way where knowledge can be delivered in a short period of time. And if we go ahead with microteaching, many topics can be covered instead of going for a seminar, which takes more time to finish. If I plan one topic, it takes more time. But if I place it in an objective-based format, I can cover the topics properly and on time.” (P11)	
		Focus on objectives	2	16.67	“PG students, who are registered nurses with practical experience in various areas before pursuing their postgraduate nursing education, benefit from microteaching by gaining a clear understanding of the evaluation criteria. They focus on these criteria while teaching, ensuring they meet the required standards. For example, one criterion may be creating an environment conducive to teaching, which they can work on effectively if they pay attention to it. This focus on specific criteria becomes a significant advantage when introducing microteaching to PG students.” (P7)	
		Recording accessibility	3	25	“This approach is really helpful for improving planning and teaching skills. Since it doesn’t allow for any mixing of unrelated content, the teacher can stay focused on one particular topic, making the session more streamlined. Another great aspect is the feedback it provides, which plays a key role in further improvement. It’s also a confidence booster for the teacher, as they can refine their delivery over time. Plus, because the sessions are short, they can easily be recorded for review or future reference.” (P12)	
Area	Physical	Classroom	10	83.33	“Classroom for the presentation, clinicals for general teaching.” (P1)	
		Clinical	8	66.66	“There are different areas where microteaching can be used, in a classroom, in clinical setup, in community, or even in our personal individual teaching or group teaching. Any setup can use microteaching. I think it will be very beneficial, mainly to improve the confidence skills of the teacher.” (P10)	
		Lab	2	16.67	“For postgraduate students, microteaching serves not only as a new and important teaching methodology but also as a tool for sharpening essential skills in class, clinical, community, and lab.” (P5)	
		Community	3	25	“Mostly, as I teach the subject community and research, I feel clinical-based teaching is important. So I prefer microteaching as well. Whenever topics have small weightage, just a few hours, I prefer to go for microteaching. In subjects like research, there are very small units or topics to be taken, like hypothesis. I want to teach them, so I prefer to go for this. It's a small topic, but lifelong learning is required when understanding must be clear. So I use microteaching also in clinical practice. When I am in the clinical setup and want to take topics like polio vaccination, or teach vaccination schedules to my students, I use microteaching. If I want to teach my students about communicable and non-communicable diseases, water sanitation processes, or research topics where both theory and practical aspects are required, then I prefer microteaching for such topics.” (P11)	
	Teaching method	Individual teaching	1	8.33	“There are different areas where microteaching can be used, in a classroom, in clinical setup, in community, or even in our personal individual teaching or group teaching. Any setup can use microteaching. I think it will be very beneficial, mainly to improve the confidence skills of the teacher.” (P10)	
		Group teaching	1	8.33	“There are different areas where microteaching can be used, in a classroom, in a clinical setup, in the community, or even in our personal individual teaching or group teaching. In any setup, you can use microteaching. I think it will be very beneficial to mainly improve the confidence skills of the teacher.” (P10)	
Skill	Practice and performance	Regular practice	5	41.67	“I believe consistent practice is essential, as incorporating it into a daily routine helps refine skills. Regular practice and demonstrations are fundamental and play a vital role in achieving improvement.” (P5)	
		Extend the time	2	16.66	“One suggestion is to extend the time allotted for microteaching and categorize students based on their understanding levels. Students can be divided into smaller groups according to their learning abilities. Grouping students by their study levels ensures effective learning and helps clarify concepts more efficiently.” (P6)	
		Skill enhancement	4	33.33	“If you ask me, there are various steps or skills you have to acquire in microteaching. A good example, if you ask me, is how you introduce the topic. There is a way to question, to explain, and to end the topic. These are various skills you have to attain in microteaching. You should know what is actually practiced during microteaching; if that is not done properly, I think you won’t be able to refine the skills of microteaching. So, as a teacher, if you ask me how you refine microteaching skills, it's better that before we tell the learner how to do microteaching, as a teacher, I have to explain how the introduction has to be done, how the questioning technique should be used. For example, a simple questioning technique we always use in microteaching, we don’t immediately throw a question to the learners. We have to pose the question, pause for a moment, then point it to them. Usually, we immediately point out and expect an answer, that’s not the way. All these things, if we teach the student-teacher properly, I think as a teacher, these are the refinements we can give learners in microteaching.” (P10)	
		Recording and review performance	2	16.66	“We can refine the skills by recording teaching sessions to review performance. Pay attention to aspects such as body language, tone, and clarity of explanations.” (P3)	
	Teaching technique	Divide into smaller groups	3	25	“Dividing students into smaller groups allows us to tailor the teaching to their needs, making it easier for them to understand. For students who grasp concepts quickly, a shorter session may suffice, while those who need more time can receive additional support.” (P2)	
		Tailored teaching needs	3	25	“Dividing students into smaller groups allows us to tailor the teaching to their needs, making it easier for them to understand. For students who grasp concepts quickly, a shorter session may suffice, while those who need more time can receive additional support.” (P2)	
		Feedback	3	25	“To improve teaching skills, it’s essential to practice more classes regularly, as this helps refine techniques and build confidence. Taking feedback is equally important because it highlights areas for improvement. By accepting and working on those gaps, teachers can enhance their effectiveness. Additionally, staying focused on the planned topic ensures that lessons remain structured and clear for the students.” (P12)	
	Communication and delivery	Body language	3	25	“We can have the record or possible videos can be taken. We can give whatever deficits or things to rectify, recommendations can be given. And even if the students want to see how they are doing while teaching, we can show the videos also. That will give better ideas to them. So, here I have to change my gestures and even facial expressions, whatever it is, so they get ideas by looking at the videos and everything. Or even when recording, they will know where to pause or where to give highlights. All these will give them ideas about what they have to work on. So through this, they can develop teaching skills.” (P8)	
		Voice modulation	3	25	“We can refine the skills by recording teaching sessions to review performance. Pay attention to aspects such as body language, tone, and clarity of explanations.” (P3)	
		Clarity of explanations	3	25	“First, we need to clearly explain what the skills are and how to execute them. If we cover this first, it will be beneficial. After that, we can move on to the demonstration. Initially, we should emphasize the importance of displaying the skills based on the theoretical knowledge they have learned. From there, we can extend the discussion further.” (P9)	

Theme I: difficulties faced

“Microteaching, as we know it, is a short teaching process. It only teaches a small portion. We face a problem when giving feedback, we can’t give concrete feedback.” (P2)

“I feel that because the time period in microteaching is very limited, we have to rush to finish the topic within that time frame.” (P3)

“The main difficulties, I think, are that it takes time to provide students with sufficient attention and to derive relevant topics. Additionally, creating and preparing audio-visual aids requires significant time and effort.” (P4)

“One major challenge in conducting microteaching for an entire syllabus is that it often becomes impractical due to time constraints. Typically, only one concept or technique can be addressed at a time, which makes the process time-consuming.” (P5)

“One of the challenges with microteaching is its short duration, typically 5 to 10 minutes, during which a single topic must be covered. For new students, this can be particularly difficult, as the limited time makes it challenging to address varying levels of understanding among students with different IQ levels. While some students may grasp the concept quickly, others may struggle due to the time constraints. Additionally, not all topics can be effectively taught using microteaching; only certain topics are suitable.” (P6)

“If you ask me this question as a teacher, if I am giving microteaching to students, definitely it is time-consuming. Secondly, if the learner does not have clarity on how microteaching should be done, he or she will not be able to perform. So, evaluating as a teacher is going to be difficult. Whether it’s the teacher or student observer, whoever is evaluating the objectives, they need to check whether all the objectives are being covered during microteaching. That, I think, is a major problem for the teacher conducting it. The main disadvantage is how the group is going to accept microteaching as a learning method.” (P10)

“The first difficulty is that the time is too short, so I’m not able to finish my topic in time, that’s one difficulty I face. Another is implementation. Sometimes I may have more knowledge about the topic, but I’m not able to share it in this short time. These are the difficulties I face.” (P11)

Theme II: benefits

“It is helpful for novice teachers to develop their skills. It helps them make their students understand things properly. Since microteaching is done in a short period, the skills are very well developed. You can use different types of techniques in that. Basically, it involves investigation, and it is like reply, observe, and again reflect back to explain the things.” (P2)

“PG students, they are already adult nurses, so for PG students, it is very useful. We just have to refine their teaching skills. So, in microteaching we do not elaborate teaching like conventional teaching for UGs. From the PG point of view, microteaching is effective. We just have to refresh their knowledge. It gives feedback and reflection.” (P3)

“I think microteaching helps teachers develop and refine their teaching skills. It allows them to recognize their strengths and areas needing improvement by identifying mistakes made during practice. This process provides a valuable opportunity for teachers to enhance their skills through targeted feedback and reflection.” (P4)

“While this can be a limitation, it also has its advantages, as it allows for in-depth focus and clarification of specific topics within any subject. Despite this difficulty, microteaching remains an effective technique for enhancing understanding.” (P5)

“For postgraduate students, the topics may be easier to understand, but for BSc students, it takes a bit more time. Therefore, the time duration for teaching should be slightly extended to accommodate their learning needs.” (P6)

“PG students, who are registered nurses with practical experience in various areas before pursuing their postgraduate nursing education, benefit from microteaching by gaining a clear understanding of the evaluation criteria. They focus on these criteria while teaching, ensuring they meet the required standards. For example, one criterion may be creating an environment conducive to teaching, which they can work on effectively if they pay attention to it. This focus on specific criteria becomes a significant advantage when introducing microteaching to PG students.” (P7)

“There are lots of benefits in microteaching. We are taking one skill and practicing it, which leads to the complete development of all skills. How to introduce the topic or how to question the students, each aspect is taken care of. How to give an explanation, how to do each and every component will be monitored and developed separately, so they gain confidence. For new learners, it helps give confidence and develop teaching skills. That is the main benefit, I can say.” (P8)

“This is basically very helpful in developing individual skills. When they go for a seminar or present a bigger topic, they can make use of all techniques they have practiced during microteaching. By this means, it helps them to practice all the techniques, for example, explanation, lecturing, introduction, probing questions. So, all the techniques or skills they have can be accumulated and articulated. They can display them altogether during a seminar, but during microteaching, they can practice each one individually and improve.” (P9)

“Basically, you are asking what the advantages of microteaching are for PG students. A major advantage is for the teacher as a beneficiary. We are not focusing on learner beneficiaries, basically, it is for teacher development. The second benefit is that the confidence of the teacher improves through microteaching. Another thing is that, if you ask about the major disadvantages, the teacher herself will personify how she should perform. All skills are developed through microteaching. Maybe when she actually teaches, she will personify all the skills, what I have to be in front of the class. That is one of the advantages. Another advantage is that teaching skills and demonstration areas will be developed. I already mentioned that confidence is one of the key areas, and that will definitely improve. Also, not only the teacher but during microteaching she will focus more on how the objectives are to be attained. When she moves toward a bigger goal, she can apply it. Microteaching is not only for teaching or demonstration, it also teaches the use of different types of AV aids for different groups of students. That is also taught during microteaching. I think these are some of the benefits learners can get.” (P10)

“The benefit is that within a short period of time we can brief students. We can also use many AV aids and innovative techniques in microteaching, which help students to better understand. It also helps because we use many AV aids, it helps students remember things for a longer time. Microteaching also helps them because students today don’t want to listen to long lectures. It’s one way to deliver knowledge in a short period of time. If we go ahead with microteaching, many topics can be covered, instead of a seminar which takes more time. If I plan one topic, it takes more time, but if I place it with an objective-based approach, I can cover the things properly and on time.” (P11)

“This approach is really helpful for improving planning and teaching skills. Since it doesn’t allow for any mixing of unrelated content, the teacher can stay focused on one particular topic, making the session more streamlined. Another great aspect is the feedback it provides, which plays a key role in further improvement. It’s also a confidence booster for the teacher, as they can refine their delivery over time. Plus, because the sessions are short, they can easily be recorded for review or future reference.” (P12)

Theme III: areas

“Classroom for the presentation, clinicals for general teaching.” (P1)

“Practical session, demonstration session, and theory part.” (P2)

“It can be used in all nursing subjects. Usually, we use it in the classroom; sometimes we even use it in hospitals as clinical teaching.” (P3)

 “Microteaching can be applied to all nursing subjects, typically used in the classroom setting. At times, it is also utilized in hospitals for clinical teaching. This approach helps in both academic and practical learning environments.” (P4)

“For postgraduate students, microteaching serves not only as a new and important teaching methodology but also as a tool for sharpening essential skills in classroom, clinical, community, and laboratory settings.” (P5)

“Classroom teaching, in theory teaching, they can also use microteaching.” (P6)

“We can use it in all teaching areas like teaching and demonstration.” (P7)

“If you take one topic, then we have to take small components of the topic. Discussing the entire topic in 20 or 15 minutes is not possible, so most of the time, only the pathophysiology of the disease condition is taken, or we take only medical management and surgical management. So only one aspect will be taken, and the topic is given to the student.” (P8)

“Basically, we are using classroom teaching because it is within a short period. We can use it in the clinical field, but it would require more time. We are doing procedures and all preparation, and it takes a little more time in comparison to classroom teaching.” (P9)

“There are different areas where microteaching can be used, in a classroom, in a clinical setup, in the community, or even in our personal individual teaching or group teaching. In any setup, you can use microteaching. I think it will be very beneficial to mainly improve the confidence skills of the teacher.” (P10)

“Mostly, as I teach the subjects community and research, I feel that clinical-based teaching is important. So, I prefer microteaching as well, especially whenever the topics have small weightage and limited hours. For example, in subjects like research, there are small units or topics to be taken, like hypothesis. I want to teach them, so I prefer microteaching. It is a small topic but requires lifelong learning with clear understanding. So, I use microteaching in clinical practice as well. When I am in the clinical setup and I want to teach topics like polio vaccination or vaccination schedules to my students, I use microteaching. In case I want to teach about communicable and non-communicable diseases, water sanitation process, or research topics that require both theory and practical aspects, I prefer microteaching for such topics.” (P11)

“There are several opportunities available for teacher development, including specialized training programs designed to enhance their skills. Clinical teaching is another effective method, offering hands-on experience in a controlled environment. Additionally, teacher training centres provide structured support and resources to help educators grow and excel in their profession.” (P12)

Theme IV: skill

“We can do practice, but the content will not be properly covered.” (P1)

“Dividing students into smaller groups allows us to tailor the teaching to their needs, making it easier for them to understand. For students who grasp concepts quickly, a shorter session may suffice, while those who need more time can receive additional support.” (P2)

“We can refine the skills by recording teaching sessions to review performance. Pay attention to aspects such as body language, tone, and clarity of explanations.” (P3)

“Students can seek feedback from peers, mentors, or students after each session. Analyze their suggestions and use them to refine your techniques.” (P4)

“I believe consistent practice is essential, as incorporating it into a daily routine helps refine skills. Regular practice and demonstrations are fundamental and play a vital role in achieving improvement.” (P5)

“One suggestion is to extend the time allotted for microteaching and categorize students based on their understanding levels. Students can be divided into smaller groups according to their learning abilities. Grouping students by their study levels ensures effective learning and helps clarify concepts more efficiently.” (P6)

“Record students' teaching sessions to assess their performance. Focus on elements such as gesture, voice modulation, and clear explanations.” (P7)

“We can have the record or possible videos can be taken. We can give whatever deficits or things to rectify, recommendations can be given. And even if the students want to see how they are doing while teaching, we can show the videos also. That will give better ideas to them. So here, I have to change my gestures and even facial expressions, whatever it is, so they get ideas by looking at videos and everything. Or even when recording, they will know where to pause or where they have to give highlights. All these give them ideas of what they have to work on. So, through this, they can develop teaching skills.” (P8)

“First, we need to clearly explain what the skills are and how to execute them. If we cover this first, it will be beneficial. After that, we can move on to the demonstration. Initially, we should emphasize the importance of displaying the skills based on the theoretical knowledge they have learned. From there, we can extend the discussion further.” (P9)

“If you ask me, there are various steps or skills you have to acquire in microteaching. A good example, if you ask me, is the way you introduce the topic. There is a way to question, how to explain, and how to end the topic. These are various skills you have to attain in microteaching. So, in all these things, you should know what is the actual practice done during microteaching. If that is not done in the proper way, I think you will not be able to refine the skills of microteaching. So as a teacher, if you ask me how you will refine microteaching skills, it's better, before we tell the learner how to do microteaching, as a teacher, I have to explain to them how the introduction has to be done, how questioning technique has to be used. For example, a simple questioning technique we always use in microteaching, we don’t immediately throw a question to the learners. It's like we have to pose the question, pause for a minute, then point out to them. See, usually what we do, we immediately point out and expect an answer. That’s not the way. All these things, if we teach the student-teacher properly, I think, as a teacher, these are the refinements we can give learners in microteaching.” (P10)

“I definitely want to say it’s a very good and innovative teaching process. If you ask me to take four or five sessions, definitely not all topics, but there are a few topics I must cover, like environmental sanitation and research areas. I need to have many microteaching sessions on these topics. And if you give me topics like gender sensitization, there I will find the skills required, like AV aids to be used properly. Even I have to have knowledge regarding time management, it’s one of the skills I require. I also need to know how to grab attention, and how to cover my topics within a short period of time.” (P11)

“To improve teaching skills, it’s essential to practice more classes regularly, as this helps refine techniques and build confidence. Taking feedback is equally important because it highlights areas for improvement. By accepting and working on those gaps, teachers can enhance their effectiveness. Additionally, staying focused on the planned topic ensures that lessons remain structured and clear for the students.” (P12)

## Discussion

Microteaching is characterized by significant challenges, primarily due to its short duration, which generally limits lesson coverage to 5 to 10 minutes. This brief time frame makes it difficult for instructors to cater to diverse student abilities and deliver comprehensive content, often leading to confusion about teaching techniques and evaluation criteria. Additionally, the extensive preparation required creates time constraints that hinder the provision of meaningful feedback. The controlled teaching environment restricts deeper engagement with topics, preventing students from fully addressing their questions. While microteaching is valuable for honing specific skills, these time limitations and content delivery issues detract from its overall effectiveness, especially in larger classes. Addressing these concerns could enhance the learning experience for both students and educators. 56% said that it frequently takes a lot of time and effort from the teacher’s side, while 44% said that it always takes time and effort from the teacher [3].

Microteaching effectively aids postgraduate nursing students in refining their teaching skills within a short timeframe, allowing for focused practice on specific techniques. It is particularly beneficial for novice teachers, helping them cultivate confidence and identify areas for improvement through targeted feedback. Unlike conventional teaching methods, microteaching concentrates on individual skills, making it easier for PG students, who already possess practical experience, to grasp evaluation criteria and enhance their teaching approaches. Additionally, the method supports the integration of various audiovisual aids, which facilitates better understanding and retention among students. Although it may require a slight extension of teaching time for BSc students, the structure of microteaching fosters clarity and allows for in-depth coverage of topics within a condensed format. Its concentrated nature enables easier recording for reflective purposes, ultimately enhancing planning and delivery skills while ensuring teachers meet their educational objectives. Most students had poor teaching skills (97.7%), but after the application, around half of them were rated as having very good teaching skills by themselves, their peers, and instructors (50%, 50%, and 48.5%, respectively). Additionally, a significant improvement in overall teaching skills was observed, with both the mean and standard deviation increasing, and the difference was statistically significant (p < 0.001) [6].

Microteaching can be effectively utilized across all nursing subjects in various settings, including classrooms and clinical environments. It serves as a valuable methodology for postgraduate students, enhancing essential teaching skills in theory and practical areas such as clinical, community, and lab settings. This approach allows for focused instruction on specific components of broader topics, facilitating deeper understanding in limited time frames. Overall, microteaching promotes teacher confidence and skill development, supported by opportunities through specialized training and clinical teaching experiences.

The absence of studies on the specific areas where microteaching skills can be applied highlights an opportunity for broader implementation. It suggests that microteaching could be effective across various nursing subjects. Both classroom and clinical settings present potential environments for its use. This opens up possibilities for enhancing teaching methods using microteaching skills and improving student engagement. Therefore, microteaching could be a valuable tool for nursing educators in diverse learning contexts.

Microteaching effectively enhances teaching skills by dividing students into smaller groups for tailored instruction. Regular practice and recorded sessions facilitate self-assessment of techniques like body language and clarity. Feedback from peers is crucial for continuous improvement. Clear explanations of skills should precede demonstrations, focusing on engagement and effective questioning. Allocating sufficient time and using audiovisual aids optimizes learning. Ultimately, consistent practice, feedback, and structured lesson planning are essential for developing confidence and teaching effectiveness.

Skills like lesson planning, presentation, explanation using examples, reinforcement, video recording, classroom management, and the effective use of audiovisual aids are essential for enhancing microteaching skills. Teachers enhance their abilities to engage learners, ask questions, manage time, and conclude lessons effectively. They also improve their class management skills, learn to select suitable activities, set teaching goals, and navigate challenges. Additionally, by observing presentations, they develop feedback evaluation skills and explore various teaching strategies [7].

The current study highlights the role of microteaching in helping educators develop and refine their teaching skills. Similarly, research on the experiences of sessional nursing teachers revealed that they found teaching rewarding, were deeply committed to their roles, and valued their clinical expertise as a key asset in education. However, many participants expressed a desire for a stronger sense of belonging within the school, with some feeling like "outsiders." Areas identified for improvement included refining systems and processes, enhancing microteaching and assessment skills, strengthening classroom management, and ensuring timely access to essential resources [1].

In the present study, microteaching is regarded not only as a significant and innovative teaching method but also as a means to refine essential skills across various settings, including classroom, clinical, community, and laboratory environments. A study on shifting images of teaching in student teachers discusses microteaching, not by analyzing the method itself, but by examining how student teachers described the activity. These descriptions reflected "images of teachers and teaching," that is, their ideas about what it means to teach and to be a teacher, both generally and personally [8].

In the present study, microteaching allows for in-depth focus and clarification on certain topics and enhances understanding. Another study investigating teachers' perspectives on learning through microteaching lessons revealed that active learning, through meaningful discussion, planning, practice, support from a knowledgeable advisor, collaborative deliberation-in-process, opportunities for trial, analysis, and revision, played a key role during teaching [9].

In this study, faculty reported that videos can be recorded, recommendations can be made, and if students wish to see how they are performing during instruction, we can show them the videos. This provides them with better insight. Therefore, I have to adjust my gestures and facial expressions, whatever they are, so they can get ideas by watching the videos and everything. A similar study revealed that one of the 22 microteaching videotapes demonstrated that students consistently struggled with how to frame the task, juggling the roles of instructor, student, classmate, and peer/friend. Microteaching, according to analysis of the tapes and questionnaires, was perceived as more similar to a “performance” or “classroom task” than actual “teaching” [10].

In the current study, faculty expressed that one of the major benefits of microteaching was the refinement of teaching skills. An international study on the use of microteaching in nursing education focused on two lecturers' experiences teaching presentation skills to student teachers using a five-step preparation process. Despite limited literature on its implementation, microteaching proved to be an effective strategy, enhancing students' confidence, communication, and teaching skills. While initial anxiety was a common challenge, structured practice and feedback helped students improve. The study provided practical guidance on implementing microteaching and emphasized its value in nurse education, highlighting the need for further support for educators to effectively integrate this methodology [11].

Microteaching provided educators with a valuable opportunity to improve their abilities through focused feedback and self-reflection, as noted in the present study. Similarly, another study examined feedback provided by preservice administrators to help preservice teachers refine their teaching skills, gain insights from experienced educators, and appreciate the value of supervision in professional growth. Additionally, the experience led to a positive shift in their perceptions of teaching, reinforcing the effectiveness of this integrated learning model [12].

In this study, microteaching plays a crucial role in building confidence among teachers, especially new learners. By focusing on individual teaching skills, such as introducing a topic, questioning students, and providing explanations, this aspect is carefully monitored and developed separately. This structured practice ensures a comprehensive improvement in teaching abilities, allowing educators to refine their techniques in a supportive environment. As a result, teachers gain the confidence needed to deliver practical lessons and continuously enhance their instructional skills. In a related study, results show that teachers developed key skills in planning, demonstration, explaining learning outcomes, assessment, and classroom management. They concluded that microteaching should be a routine practice in schools, as it boosts confidence, enhances skills, and promotes the sharing of teaching methods [13].

In our study, microteaching helps teachers develop and refine their teaching skills. Similarly, one study showed that self-assessments and observations revealed that teachers who received structured video feedback practiced significantly more targeted teaching behaviors than those in the control group. These findings highlight the power of domain-specific video feedback in enhancing teaching quality [14].

A major limitation of the current study was the lack of sufficient research evidence available during the literature review, which may have impacted the depth of contextual analysis and comparison with existing studies. Despite the strengths of this study, several limitations must be acknowledged. Firstly, the qualitative nature of the research, particularly the phenomenological design, limits the generalizability of the findings beyond the selected nursing colleges in Mumbai and Navi Mumbai. The relatively small sample size of 12 (100%) post-graduate nursing teachers, though sufficient for in-depth exploration, may not fully capture the diverse perspectives and experiences of nursing educators across different institutions. Additionally, the use of simple random sampling by the lottery method, while ensuring fairness, might not account for variations in teaching methodologies and institutional policies that could influence the use of microteaching. The reliance on self-reported data through face-to-face interviews introduces the possibility of response bias, as participants may provide socially desirable answers rather than fully candid reflections. Furthermore, the exclusion of teachers with formal certification or PG diplomas in education, while ensuring a focus on those without specialized training, may limit insights into how structured educational training impacts perceptions of microteaching. Finally, while audio recordings were used to maintain data accuracy, the potential for interviewer influence or misinterpretation during qualitative analysis cannot be entirely ruled out. These limitations suggest the need for further research with a larger and more diverse sample, incorporating mixed-method approaches to enhance the depth and applicability of the findings. The study was limited to post-graduate nursing faculty with three years of experience.

## Conclusions

Microteaching faces challenges due to its short duration, typically limiting lessons to 5-10 minutes, which restricts coverage and makes it difficult to address diverse student needs. The brief timeframe can lead to confusion regarding teaching methods and evaluation standards, while preparation demands can limit the quality of feedback. Additionally, the controlled teaching environment prevents in-depth engagement with topics. However, microteaching is valuable for developing specific teaching skills, particularly for novice educators and postgraduate students. Its focused approach supports the use of audiovisual aids, encourages self-reflection, and fosters more efficient lesson planning and delivery. There is significant potential for the application of microteaching skills across all nursing subjects, both in classroom and clinical settings.

## Appendices

Appendix 1

**Table 3:** Tool used for data collection.

Section	Questions
Section I: Demographic data	Age
	Gender
	Qualification
	Specialty
	Designation
	Years of teaching experience after post-graduation
	Years of teaching experience to post graduate students
	Subjects taught
Section II: Open-ended questionnaire	What are the difficulties you face while using microteaching as a teaching technique?
	What are the benefits of use of microteaching in teaching PG students?
	Which are the areas used for microteaching?
	How will you refine the skills of microteaching?
